# Supplementary material for: DMRforPairs: identifying Differentially Methylated Regions between unique samples using array based methylation profiles
Source: BMC Bioinformatics. 2014 May 15;15:141. doi: 10.1186/1471-2105-15-141 (PMC4046028; doi:10.1186/1471-2105-15-141)
Supplement: Additional file 2 — DMRforPairs output for the comparison of A431-MCF7 and NA17018-NA17105. Please start from the HTML files in each folder. Available via the BMC Bioinformatics website. [file 1471-2105-15-141-S2.zip › 1394847754114233_MOESM2_ESM/A431_MCF7/figures/29116.pdf]

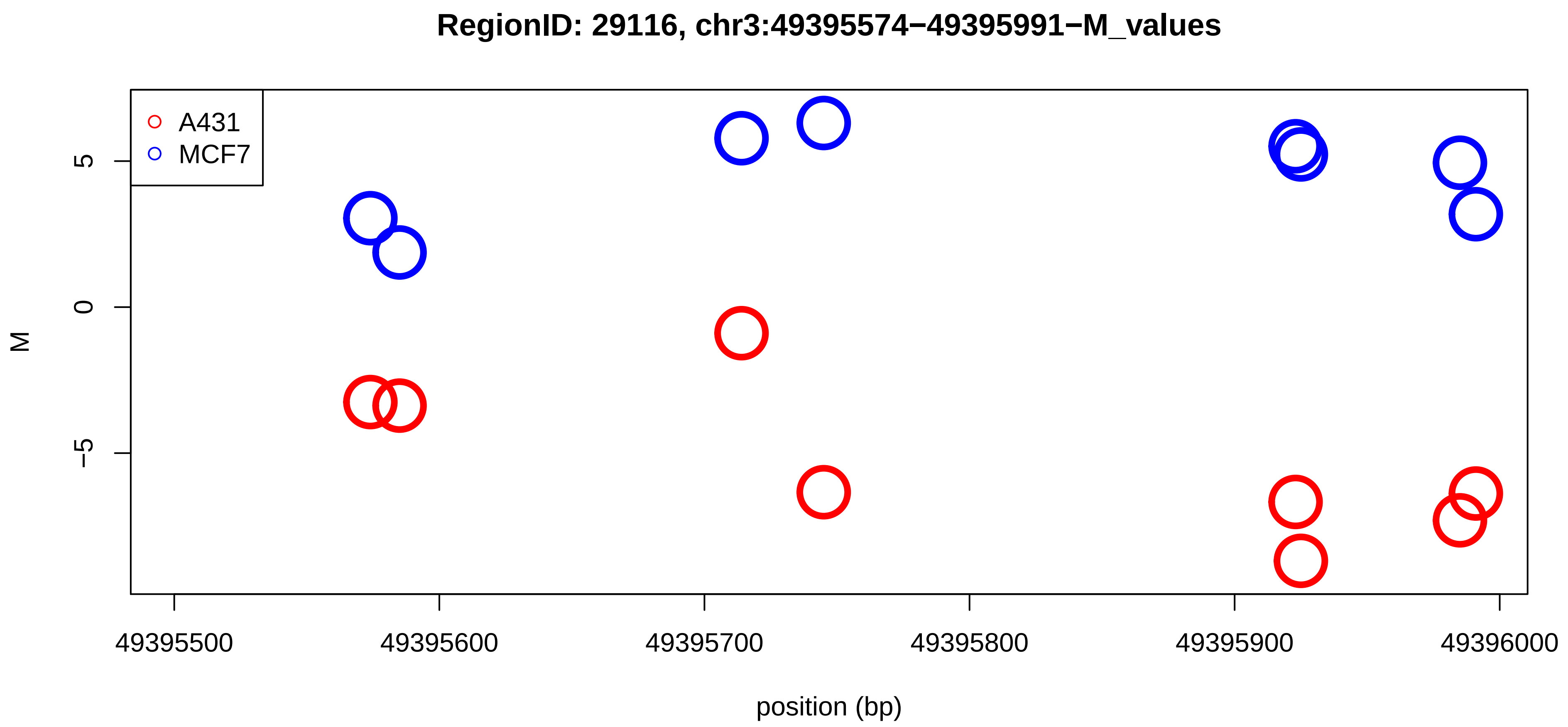
RegionID: 29116, chr3:49395574−49395991−M_values
A431
MCF7
M
5
0
−5
49395500
49395600
49395700
49395800
49395900
49396000
position (bp)

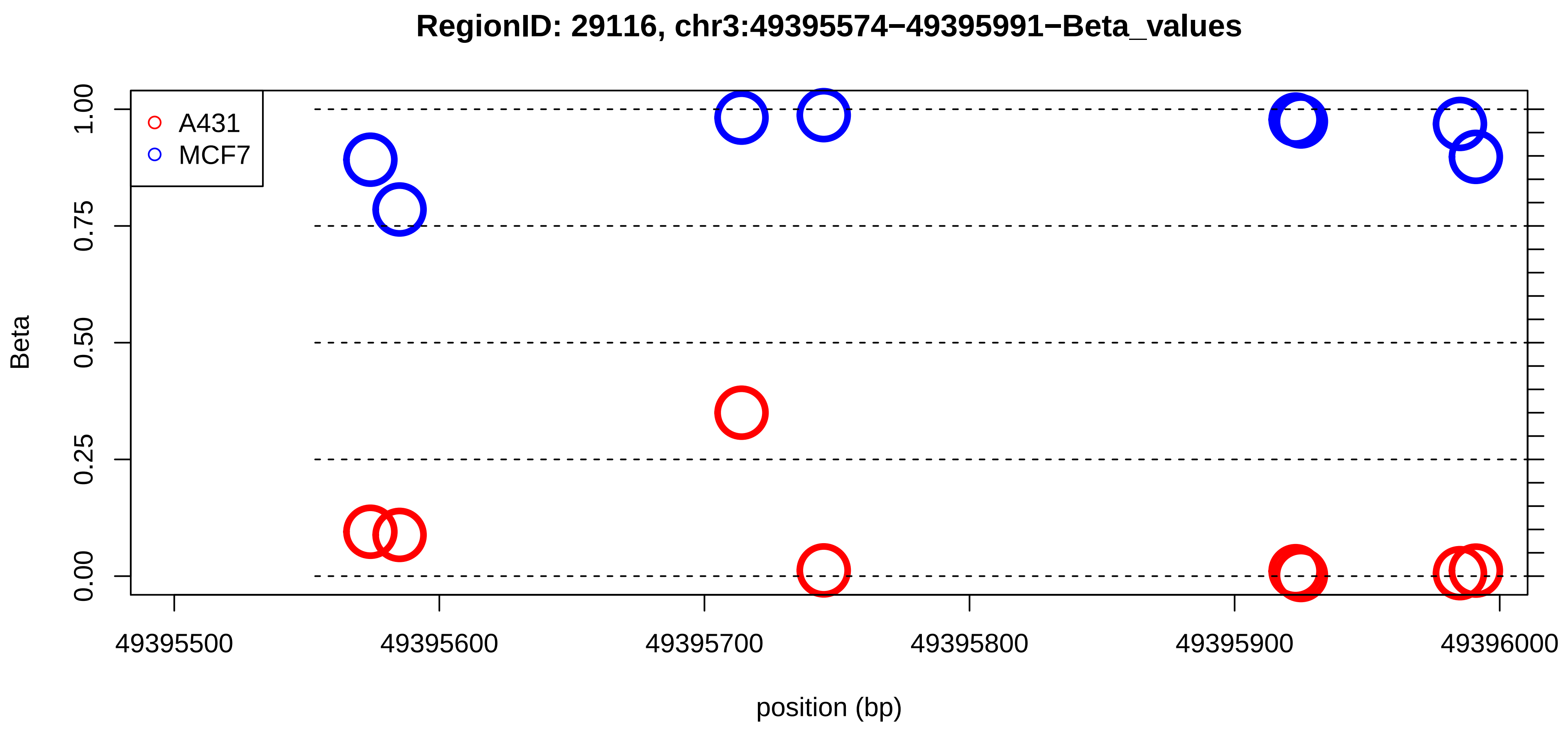
RegionID: 29116, chr3:49395574−49395991−Beta_values
A431
MCF7
Beta
1.00
0.75
0.50
0.25
0.00
49395500
49395600
49395700
49395800
49395900
49396000
position (bp)

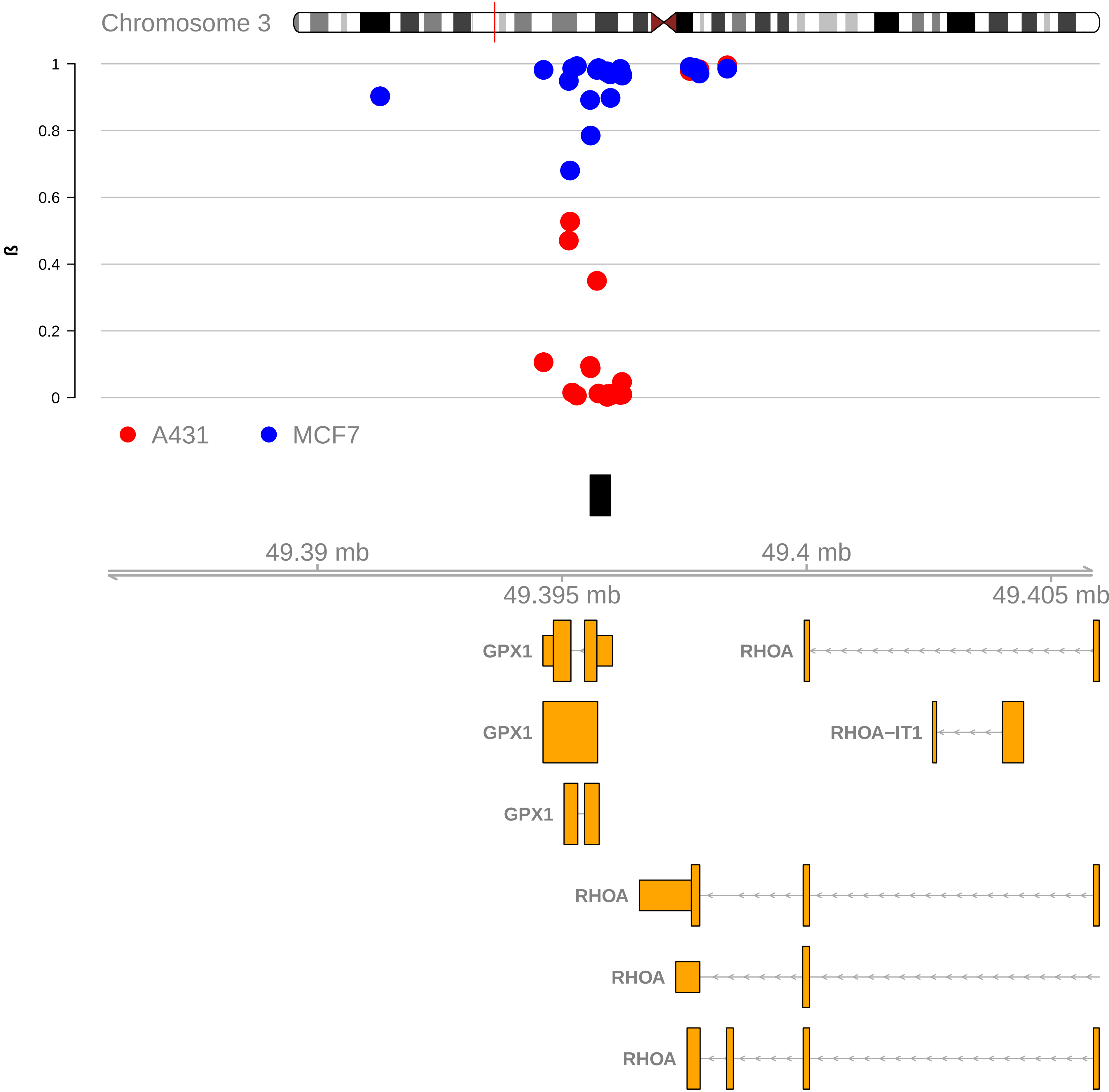
Chromosome 3
1
0.8
0.6
0.4
0.2
0
ß
A431
MCF7
49.39 mb
49.4 mb
49.395 mb
49.405 mb
GPX1
RHOA
GPX1
RHOA−IT1
GPX1
RHOA
RHOA
RHOA
